# Using Graph Attention Network and Graph Convolutional Network to Explore Human CircRNA–Disease Associations Based on Multi-Source Data

**DOI:** 10.3389/fgene.2022.829937

**Published:** 2022-02-07

**Authors:** Guanghui Li, Diancheng Wang, Yuejin Zhang, Cheng Liang, Qiu Xiao, Jiawei Luo

**Affiliations:** ^1^ School of Information Engineering, East China Jiaotong University, Nanchang, China; ^2^ School of Information Science and Engineering, Shandong Normal University, Jinan, China; ^3^ College of Information Science and Engineering, Hunan Normal University, Changsha, China; ^4^ College of Computer Science and Electronic Engineering, Hunan University, Changsha, China

**Keywords:** circRNA-disease associations, deep learning, graph attention network, graph convolutional network, centered kernel alignment

## Abstract

Cumulative research studies have verified that multiple circRNAs are closely associated with the pathogenic mechanism and cellular level. Exploring human circRNA–disease relationships is significant to decipher pathogenic mechanisms and provide treatment plans. At present, several computational models are designed to infer potential relationships between diseases and circRNAs. However, the majority of existing approaches could not effectively utilize the multisource data and achieve poor performance in sparse networks. In this study, we develop an advanced method, GATGCN, using graph attention network (GAT) and graph convolutional network (GCN) to detect potential circRNA–disease relationships. First, several sources of biomedical information are fused *via* the centered kernel alignment model (CKA), which calculates the corresponding weight of different kernels. Second, we adopt the graph attention network to learn latent representation of diseases and circRNAs. Third, the graph convolutional network is deployed to effectively extract features of associations by aggregating feature vectors of neighbors. Meanwhile, GATGCN achieves the prominent AUC of 0.951 under leave-one-out cross-validation and AUC of 0.932 under 5-fold cross-validation. Furthermore, case studies on lung cancer, diabetes retinopathy, and prostate cancer verify the reliability of GATGCN for detecting latent circRNA–disease pairs.

## Introduction

Circular RNA (circRNA) is a novel endogenous non-coding RNA forming a covalently closed loop structure, which lacks a 50-end cap and a 30-end ployA tail (Memczak et al., 2013; Meng et al., 2017). This structure is beneficial for circRNA to develop resistance to RNA exonuclease degradation and provides a more stable biological expression (Li et al., 2015). As a result, in most species, the average half-life of circRNAs is substantially increased than their linear equivalent. When circRNAs were first found as early as 1970s, they had been regarded as the abnormal shear or product of “shear noise,” limited to the level of technology and knowledge at that time. In previous studies, multiple circRNAs were verified to be widespread in eukaryotes and play an essential role in biological functions with the advancement of biology and sequencing technologies. Currently, the biological functions of circRNA are reflected as follows (Rong et al., 2017): regulation of alternative splicing or transcription, miRNA sponges, regulation of protein binding, and generation of pseudogenes.

CircRNA has become a new biomarker due to its abundance, structural stability, developmental stage specificity, and tissue specificity (Zhang Z. et al., 2018), which can be discovered in saliva, blood, and exosomes. Cumulative research studies have confirmed that multiple circRNAs are significant to the expression of various pathological conditions (Han et al., 2018; Zhu et al., 2017; Zhang S. et al., 2018), especially cancer (Vo et al., 2019), cardiovascular, cerebrovascular, and nervous system diseases. For instance, circRNA hsa_circ_0027599 is overexpressed in gastric cancer (Wang L. et al., 2018), thereby regulating the expression of the gene PHLDA1 and promoting tumorigenesis. In cardiovascular and cerebrovascular diseases, circRNA circWDR77Z targets and regulates miRNA miR-124/FGF-2 through the “sponge” function (Chen et al., 2017), which affects the migration and proliferation for vascular smooth muscle cells, thereby promoting atherosclerosis development. For myocardial infarction, overexpression of circRNA CDR1 leads to the upregulation of downstream corresponding enzymes and proteins (Zhang et al., 2016), thereby aggravating myocardial infarction. In neurological diseases, the expression of circRNA in brain tissue is different, and its distribution in the brain is uneven (Zhang et al., 2021b).

Although circRNA is commonly expressed in various cell lines and tissues with strong tissue specificity and development stage specificity, the pathogenic mechanism of circular RNA and how it interacts with other biological molecules remain unknown. In recent years, researchers have established many experimentally verified or reported databases on relationships between circRNAs and diseases, such as circBase (Glažar et al., 2014), circRNADb (Chen et al., 2016), circR2Disease (Fan et al., 2018b), circRNADisease (Zhao et al., 2018), circ2Disease (Yao et al., 2018) and circ2Traits databases (Ghosal et al., 2013). Considering that conventional biological studies are cost-ineffective and time-consuming, several computational approaches have been designed to detect relationships between diseases and circRNAs efficiently (Xiao et al., 2022; Lei et al., 2021). At present, the proposed computational models for discovering relationships between diseases and circRNAs are mainly classified into the following groups:

Network propagating methods have been widely applied to detect correlations between diseases and various biological entities, including circRNAs, due to the efficient use of network structure information (Peng et al., 2018). Zhang et al. designed a linear neighbor marker propagation approach named CD-LNLP *via* neighbor similarity to reveal relationships between diseases and circRNAs (Zhang et al., 2019). Li et al. presented the DWNCPCDA using DeepWalk and network consistency projection (Chen et al., 2018) to detect unobserved associations between diseases and circRNAs (Li G. et al., 2020). Lei et al. constructed a prediction model named RWRKNN, which combined the k-nearest neighbor and RWR to calculate weighted features for diseases and circRNAs (Lei and Bian, 2020).

Path-based methods are widely adopted to calculate potential interactions between diseases and circRNAs by measuring the weight of paths in different networks. Lei et al. presented a path-weighted method named PWCDA, which predicted the circRNA–disease relationships by calculating the probability value for each circRNA–disease pair *via* path information (Lei et al., 2018). Fan et al. presented the model named KATZHCDA *via* the circRNA expression profile, the similarity of the disease phenotype, and the nuclear similarity of the Gaussian interaction profile using the KATZ method to detect potential interactions between diseases and circRNAs through the heterogenous network (Fan et al., 2018a). Zhao et al. revealed a computed method named IBNPKATZ using the bipartite network projection model and the KATZ (Zhang et al., 2021a) model to discover circRNA–disease interactions (Zhao et al., 2019).

Matrix factorization–based methods have been carried out for detecting circRNA–disease relationships by constructing a low-dimensional matrix to represent the initial input features (Wang P. et al., 2018; Peng et al., 2020a). Wei et al. used weight-based nearest neighbor nodes to reconstruct the association matrix and designed a graph regularized non-negative matrix factorization algorithm iCircDA-MF to detect relationships between diseases and circRNAs (Wei and Liu, 2020). Lu et al. constructed a model named DMFCDA with deep matrix factorization, which infers potential circRNA–disease interactions by mapping features of diseases and circRNAs into low-dimensional spaces (Lu et al., 2021). Yan et al. used the Kronecker product kernel to design a regularized least squares algorithm called DWNN-RLS to detect relationships (Yan et al., 2018). Li et al. presented an advanced approach named SIMCCDA by regarding predicting associations as a recommendation system task, which achieves outstanding performance for discovering circRNA–disease associations (Li M. et al., 2020).

Deep learning integrates low-level features to construct high-level representations of features or attribute categories through the deep non-linear network structure (Peng et al., 2021; Zhou et al., 2021). Wang et al. designed a model to reveal interactions between diseases and circRNAs using deep convolutional neural networks and deep generative adversarial networks (Wang et al., 2020a). Wang et al. designed an approach named GCNCDA to identify disease-related circRNAs, which extracts high-level features contained in the circRNA–disease heterogenous network through graph convolutional networks to calculate association scores (Wang et al., 2020b). GATCDA is a novel model for discovering the correlation between diseases and circRNAs, which learns the latent representation of nodes by assigning corresponding weights to each neighbor node (Bian et al., 2021). Xiao et al. designed a computational model named NSL2CD that adopts network embedding by adaptive subspace learning (Xiao et al., 2021).

Although the abovementioned approaches have achieved excellent predictive performance, there are still several limitations given as follows: First, network-based methods achieve poor performance in sparse networks due to a small amount of network structure information. Second, path-based methods fail to dynamically calculate weights based on known associations, which makes it unable to efficiently detect relationships between diseases and circRNAs with new diseases or circRNAs. Third, matrix factorization–based methods could not discover a non-linear interaction between diseases and circRNAs. Last, current deep learning–based methods could not effectively utilize the multisource data and only pay more attention to features of the neighbor nodes or the node itself, respectively.

To solve the abovementioned challenges, we develop an advanced method GATGCN *via* graph attention network (GAT) and graph convolutional network (GCN) to detect potential circRNA–disease relationships. The complete process could be summarized as four steps: First, multisource similarity data for circRNAs and diseases are fused by the centered kernel alignment model (CKA) (Cristianini et al., 2006). Second, we adopt the graph attention network to learn the dense representation of nodes on fused disease similarity network and fused circRNA similarity network. Third, we construct the heterogenous network by connecting circRNA–disease interaction network, feature matrix of diseases, and feature matrix of circRNAs. Finally, the graph convolutional network is adopted to get prediction scores based on the heterogenous network. According to reliable computer experiments, GATGCN outperforms several state-of-the-art methods with a prominent AUC of 0.932.

## Materials

### Human CircRNA–Disease Associations

The circR2Disease provides verified relationships between diseases and circRNAs, which is a manually curated database including 739 known relationships between 100 diseases and 676 circRNAs. We eventually extract 661 associations between 88 diseases and 585 circRNAs for humans after removing the associations unrelated to human species and duplicate associations.

### Human Disease–MiRNA Associations

MiRNAs are significant to pathogenesis and treatment of diseases as the important regulatory molecule for genes. On dataset, we collect 1,883 experimentally verified disease–miRNA relationships between 462 miRNAs and 88 diseases from the HMDD (Li et al., 2014), which provides disease-associated miRNAs and their target genes, including 8,802 known relationships between 350 diseases and 32281 miRNAs.

### Human Disease–Gene Associations

Due to gene mutation and expression affecting diseases, diseases are closely related to genes. On the dataset, 74 experimentally verified disease–gene associations between 61 genes and 88 diseases are filtered out, downloaded from http://cssb2.biology.gatech.edu/knowgene/.

### Human CircRNA–MiRNA Associations

With plenty miRNA binding sites (Hansen et al., 2013; Peng et al., 2020b), circRNAs actively affect the expression of miRNA’s downstream genes as miRNA sponges (Peng et al., 2017; Zeng et al., 2020). We obtain 17844 known circRNA–miRNA associations between 640 miRNAs and 585 circRNAs from ENCORI (available at http://starbase.sysu.edu.cn/agoClipRNA.php? source=circRNA).

### Human CircRNA–Gene Associations

According to the previous research, circRNAs are verified to be significant in regulating the expression of genes. On the dataset, 487 known circRNA–gene associations between 418 genes and 585 circRNAs are extracted from http://cssb2.biology.gatech.edu/knowgene/search.html.

### Disease Semantic Similarity

The semantic information of the diseases has been wildly adopted to measure the similarity of diseases because of its effectiveness and stability. In this study, we obtain the related annotation terms for each disease from MeSH.

In MeSH, the directed acyclic graph (DAG) is applied to represent the semantic relationship among diseases, in which nodes denote corresponding disease information and directed edges denote the relationship among diseases. Specifically, disease *d*_*i*_ can be described as three items *DAG*_*i*_ = [*d*_*i*_, *T* (*d*_*i*_, *E*(*d*_*i*_))], where *T*(*d*_*i*_) represents *d*_*i*_ itself and its ancestor nodes and *E*(*di*) is relationships between *d*_*i*_ and all diseases. The contribution of disease *d*_*i*_ in *DAG*_*i*_ is formulated as follows:

$$\{\begin{array}{c} D_{d_{i}}\left(n\right)=1\;\mathrm{if}\;n=d\\ D_{d_{i}}\left(n\right)=\max\left\{\sigma \cdot D_{d_{i}}\left(n\prime \right)|n\prime \in children\;of\;n\right\}\;\mathrm{if}\;n\neq d \end{array},$$ (1)

where *σ* denotes the attenuation factor for semantic contribution, which is defined as the optimal value of 0.5 according to Wang’s experience Wang et al. (2010); *n'* represents the child node of the node *n*. Therefore, the overall semantic score of the disease *d*_*i*_ is measured by accumulating the contribution scores from its ancestor diseases and itself as follows:

$$D\left(d_{i}\right)=\sum_{n\in T\left(d_{i}\right)}D_{d_{i}}\left(n\right).$$ (2)

In general, diseases with more common parts shared in the DAG achieve higher semantic similarities. Based on this hypothesis, the value of disease semantic similarity between disease *d*_*i*_ and disease *d*_*j*_ is formulated *via* Eq.3:

$$DS\left(d_{i},d_{j}\right)=\frac{\sum_{n\in T_{d_{i}}\cap T_{d_{j}}}\left(D_{d_{i}}\left(n\right)+D_{d_{j}}\left(n\right)\right)}{D\left(d_{i}\right)+D\left(d_{j}\right)}.$$ (3)

### CircRNA Functional Similarity

According to previous studies, circRNAs that are relevant to more similar diseases are prone to be more similar in functions (Li et al., 2019). Then, the BMA method is deployed to measure the functional similarity score among different circRNAs according to relevant disease sets. Given a specific disease *d*_*i*_ and *D* = (*d*_1_, *d*_2_, … , *d*_*t*_), the score of functional similarity between circRNA *c*_*i*_ and circRNA *c*_*j*_ is measured *via* Eqs 4, 5:

$$FS\left(c_{i},c_{j}\right)=\frac{\sum_{m=1}^{\left|D_{i}\right|}S\left(d_{m},D_{j}\right)+\sum_{n=1}^{\left|D_{j}\right|}S\left(d_{n},D_{i}\right)}{\left|D_{i}\right|+\left|D_{j}\right|},$$ (4)

$$S\left(d_{m},D_{j}\right)=\underset{1\leq t\leq |D_{j}|}{\max}\left(S\left(d_{m},d_{t}\right)\right),$$ (5)

where *D*_*j*_ represents the collection of diseases associated with circRNA *c*_*j*_. *S*(*d*_*m*_, *D*_*j*_) represents the similarity between disease *d*_*m*_ associated with circRNA *c*_*i*_ and disease collection *D*_*j*_ associated with circRNA *c*_*j*_.

### Pearson’s Correlation Coefficient Similarity

Since the circRNA functional similarity network and the disease semantic similarity network are prone to be sparse, we adopt Pearson’s correlation coefficient approach to enrich multisource similarity data by calculating the linear correlation among different variables. To be specific, the value of Pearson’s correlation between variable *M* and variable *N* is measured as follows:

$$\mathrm{Cor}\left(M,N\right)=\frac{\mathrm{cov}\left(M,N\right)}{\sqrt{\mathrm{var}\left(M\right)\mathrm{var}\left(N\right)}},$$ (6)

where *var*(*M*) measures the variance of *M*; *cov*(*M*, *N*) calculates the covariance between *M* and *N*; the value of *Cor*(*M*, *N*) ranges from −1 to 1, which reflects the strength of the linear correlation between *M* and *N*.

Four binary networks have been built including the disease–gene network, circRNA–miRNA network, circRNA–gene network, and disease–miRNA network. Then, Pearson’s correlation coefficient approach is adopted to compute disease similarity and circRNA similarity *via* corresponding bipartite networks. The equation is computed as follows:

$$\mathrm{Cor}\left(n_{i},n_{j}\right)=\frac{\mathrm{cov}\left(\mathrm{IP}\left(n_{i}\right),\mathrm{IP}\left(n_{j}\right)\right)}{\sqrt{\mathrm{var}\left(\mathrm{IP}\left(n_{i}\right)\right)\mathrm{var}\left(\mathrm{IP}\left(n_{j}\right)\right)}},$$ (7)

where *IP*(*n*_*i*_) denotes the *i*th row of the corresponding association network. *Cor*(*n*_*i*_, *n*_*j*_) denotes the value of Pearson’s correlation similarity between node *n*_*i*_ and node *n*_*j*_ based on the corresponding association network.

## Methods

**FIGURE 1:**
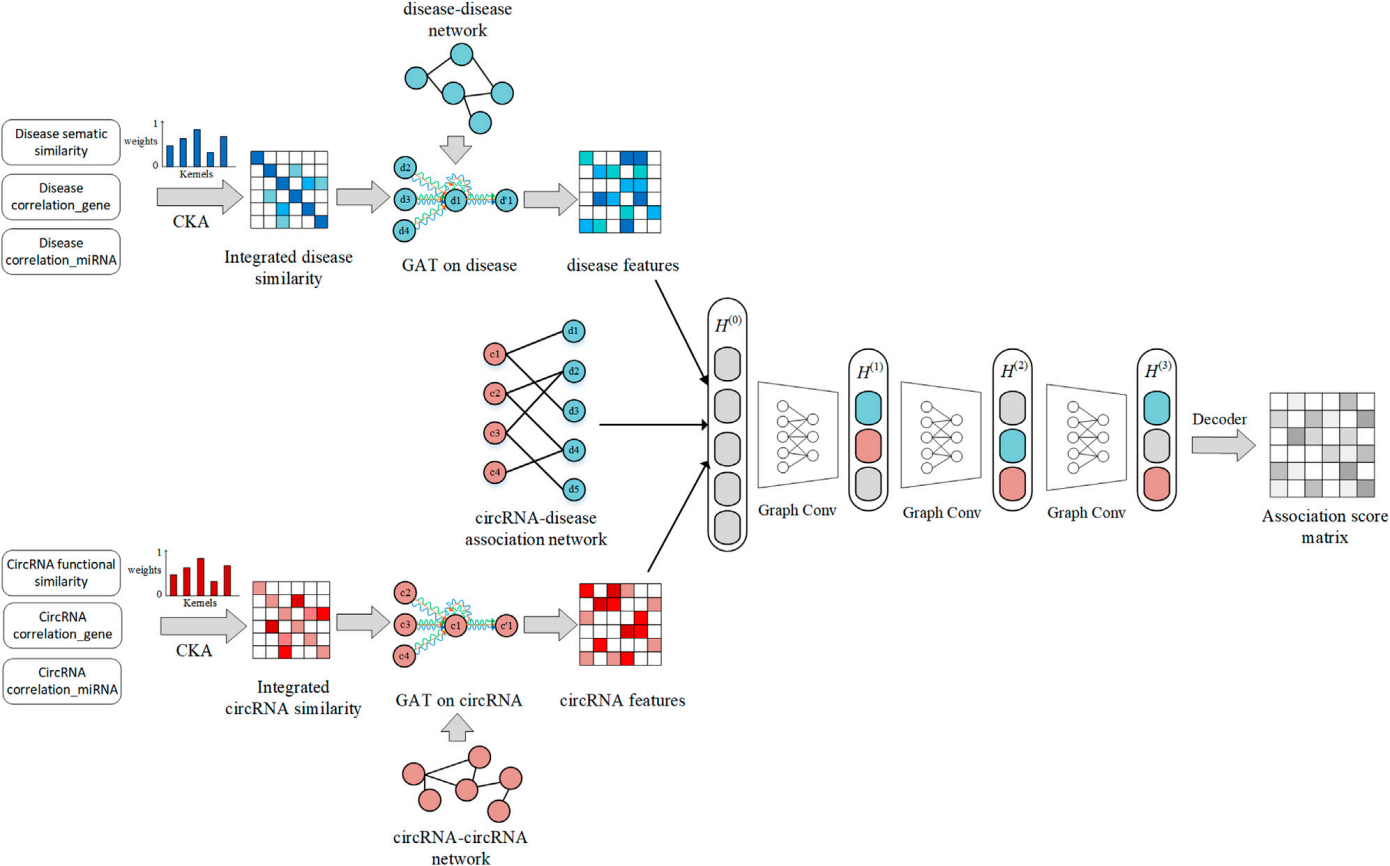
Overall workflow of the GATGCN.

In this work, we develop an advanced method GATGCN *via* the graph attention network and graph convolutional network to detect potential circRNA–disease relationships. As shown in Figure 1, the complete process could be summarized in four steps: First, the CKA-based model is adopted to fuse multisource similarity data for circRNAs and diseases. Second, we adopt the graph attention network to calculate the dense representation of nodes on the fused disease similarity network and fused circRNA similarity network. Third, we construct the heterogenous network, including circRNA–disease interactions network, feature matrix of diseases, and feature matrix of circRNAs. Eventually, the graph convolutional network is adopted to get prediction scores based on the constructed heterogenous network.

### Centered Kernel Alignment

In previous studies, multisource data are usually fused by calculating the average value, which ignores the importance among different kernels. Thus, the centered kernel alignment (CKA) model (Wang et al., 2021) is adopted to fuse several kinds of similarities for diseases and circRNAs based on different weights. We consider *K*_*d*_ = {*K* ^*1*^_*d*_, …, *K* ^*v*^_*d*_} and *K*_*c*_ = {*K* ^*1*^_*c*_, …, *K* ^*u*^_*c*_} as different kernels for disease space and circRNA space. The *v* and *u* denote the number of kernels from disease space and circRNA space, respectively. Meanwhile, the basic CKA model (Cristianini et al., 2006) is used as the objective of MKL (Ding et al., 2019) to measure the corresponding weight of each kernel.

To be specific, the kernels *K* ^∗^_*c*_ and *K* ^*^_d_ based on optimal weight are calculated as follows:

$$K_{c}^{*}=\sum_{p=1}^{u}a_{c}^{p}K_{c}^{p},\;K_{c}^{p}\in R^{m\times m},$$ (8)

$$K_{d}^{*}=\sum_{p=1}^{v}a_{d}^{q}K_{d}^{q},\;K_{d}^{q}\in R^{n\times n},$$ (9)

where *ɑ*_*c*_ = {*ɑ* ^1^_*c*_, …, *ɑ* ^*u*^_*c*_}and *ɑ*_*d*_ = {*ɑ* ^1^_*d*_, …, *ɑ* ^*v*^_*d*_}.

Basic CKA (Cristianini et al., 2006) is adopted to calculate the weights of each kernel on the training set. The kernel alignment score between the two kernels is formulated as follows:

$$U\left(E,I\right)=\frac{{\langle E,I\rangle }_{F}}{{\left\|E\right\|}_{F}{\left\|I\right\|}_{F}},$$ (10)

where *E*, *I* denotes the corresponding similarity matrix, ||*E|*|_*F*_ denotes the Frobenius norm, and <*E*, *I*> = *Trace*(*E* ^*T*^ *I*) denotes the Frobenius inner product. The kernel alignment score represents the similarity among different kernels. Specifically, the kernel alignment score between the similarity kernel (disease kernel or circRNA kernel) and the ideal kernel matrix is measured as follows:

$$\underset{\beta \geq 0}{\max}CU\left(K^{*},K_{ideal}\right)=\underset{\beta \geq 0}{\max}\frac{{\langle Z_{N}K^{*}Z_{N},K_{ideal}\rangle }_{F}}{{\left\|Z_{N}K^{*}Z_{N}\right\|}_{F}{\left\|K_{ideal}\right\|}_{F}},$$ (11)

$$subject\text{˜}to\text{˜}K^{*}=\sum_{p=1}^{N}\beta^{p}K^{p}\;\beta \geq 0,p=1,2...,N,$$ (12)

$$\sum_{p=1}^{N}\beta^{p}=1,$$ (13)

where *K*_*ideal*_ denotes a label kernel constructed by known associations; *K*_*ideal*,__*d*_ = Y^*T*^_*train*_Y_*train*_ ∈ *R* ^*n*×*n*^ and *K*_*ideal*,__*c*_ = Y_*train*_Y^*T*^_*train*_ ∈ *R* ^*m*×*m*^ denote the ideal kernel of diseases and circRNAs, respectively.

### Attention Mechanism on Similarity

Considering that current methods did not capture potential features on the similarity network, we adopt the graph attention method to learn latent representation of diseases and circRNAs, which assigns corresponding weights to different node features based on the local graph structure to ignore noise and redundancy. The advantage of the attention mechanism is to directly evaluate which features are preferred embedding for specific downstream tasks by calculating the weights. First, we obtain the corresponding association matrix by setting a threshold on the similarity network for diseases and circRNAs. Then, the GAT (Veličković et al., 2017) is applied to learn dense representation for diseases and circRNAs as follows:

The input layer of the graph attention network is formulated as follows:

$$f=\left\{f_{1},f_{2},...,f_{N}\right\},f_{i}\in R^{F},$$ (14)

where *F* denotes the dimension of features, and *N* represents the number of nodes in the corresponding similarity network. *f* ∈ *R* ^*N*×*F*^ is constructed by the features of nodes in the corresponding similarity network. The output layer of the graph attention network is defined as follows:

$$f^{\prime }=\left\{f_{1}^{\prime },f_{2}^{\prime },...,f_{i}^{\prime }\right\},f_{i}^{\prime }\in R^{F\prime },$$ (15)

where *F′* denotes the length of learned features, and *f'* ∈ *R* ^*N*×*F'*^ represents the learned latent representations of nodes in the network. The first step is to calculate the weight of the corresponding neighbor node. The importance of the given nodes is computed by the self-attention mechanism. For each association pair between node *n*_*i*_ and node *n*_*j*_, the attention coefficient *e*_*ij*_ is calculated as follows:

$$e_{ij}\left(n_{i},n_{j}\right)=att\left(Wf_{i},Wf_{j}\right),$$ (16)

where *att* represents a mapping function transforming high-level features to a real number for association pair between node *n*_*i*_ and node *n*_*j*_ based on input features, and *W* ∈ *R* ^*F'*×*F*^ denotes a trainable weight matrix. To avoid the influence of dimension between different attention coefficients, *e*_*ij*_ is further normalized as follows:

$$\theta_{ij}=\mathrm{sof}t\max\left(e_{ij}\right)=\frac{\exp\left(e_{ij}\right)}{\sum_{t\in N_{i}}\exp\left(eit\right)\prime },$$ (17)

where *N*_*i*_ represents the collection of neighbor nodes of node *n*_*i*_. *θ*_*ij*_ denotes the normalized weight representing the importance between node *n*_*i*_ and node *n*_*j*_ in the network.

From the abovementioned formula, we obtain the combined attention mechanism as follows:

θij=exp(leakyRelu(aT[Wfi||Wft]))∑t∈Niexp(leakyRelu(aT[Wfi||Wft]))′, (18)

where *leakyRelu* denotes a non-saturated activation function, which can solve the vanishing gradients and accelerate convergence. *a* ∈ *R* ^2*F'*^ denotes the weight matrix, which maps features to a real number. The second step is to aggregate the features of all neighbors for a given node by integrating the corresponding weight. The aggregation between the given node and neighbors is formulated as follows:

$$f_{i}^{\prime }=\sigma \left(\sum_{t\in N_{i}}\theta_{it}Wf_{t}\right)$$ (19)

where *σ* denotes a non-saturated activation function. Multi-head attention mechanism is applied in GAT to integrate features and prevent overfitting. The output with the multi-head attention mechanism contains the features in different representation subspaces, which enhances the expressive capacity of the model. To be specific, the multi-head attention model based on the combination of K-independent attention mechanisms learns latent features as follows:

$$f_{i}^{\prime }=\sigma \left(\frac{1}{K}\sum_{K=1}^{K}\sum_{t\in N_{i}}\theta_{it}^{k}\cdot W^{K}f_{t}\right),$$ (20)

where *K* represents the number of self-attention models. *W* ^*k*^ denotes the trained weight matrix of the *k*th attention model.

### Heterogenous Network

The heterogenous network is constructed as initial features of GCN, including circRNA–disease associations, learned feature matrix of circRNAs, and learned feature matrix of diseases. The binary matrix *A* is constructed, and *A*_*ij*_ = 1 if the interaction between circRNA *c*_*i*_ and disease *d*_*j*_ has been verified; otherwise *A*_*ij*_ = 0. The learned feature matrix of circRNAs and learned feature matrix of diseases based on GAT are denoted as matrix *S*_*c*_ and matrix *S*_*d*_, respectively. The heterogenous network *A*_*H*_ is defined as follows:

$$A_{H}=[\begin{array}{c} S^{c}\\ A^{T} \end{array}\;\begin{array}{c} A\\ S^{d} \end{array}]\in R^{\left(M+N\right)\times \left(M+N\right)}.$$ (21)

### Graph Convolutional Network on Heterogenous Network

In recent years, GCN has achieved superior performance in node prediction, node classification, and edge prediction tasks (Kipf and Welling, 2016). In order to discover potential relationships between diseases and circRNAs, GCN models (Wang et al., 2020b) are designed to effectively extract features of circRNA–disease relationships based on the global graph structure by aggregating feature vectors of neighbors. To be specific, given a network *G*, each layer of the GCN model embedding is formulated as follows:

$$H^{\left(l+1\right)}=\sigma \left(D^{-\frac{1}{2}}GD^{-\frac{1}{2}}H^{\left(l\right)}W^{\left(l\right)}\right),$$ (22)

where *H* ^(*l*)^ denotes the propagation of features at the *l*th layer, σ(·) represents a nonlinear activation function, *D* = diag( $\underset{i}{\sum }G_{ij}$ ) denotes the degree matrix of *G*, and *W* ^(*l*)^ is the trained weight matrix at the *l*th layer. GCN integrates low-level features to construct high-level representations of nodes on the constructed heterogenous network *A*_*H*_. In addition, we adjust the number of graph convolutional network layers and set node dropout to avoid overfitting, which can reduce excessive parameters and improve the generalization ability of the GATGCN. The penalty factor *µ* is set to regulate the contribution of learned similarity features in the embedding of graph convolutional layers. Specifically, the input heterogenous network *G* is defined as follows:

$$G=[\begin{array}{c} \mu \cdot S^{c}\\ A^{T} \end{array}\;\begin{array}{c} A\\ \mu \cdot S^{d} \end{array}].$$ (23)

Then, the initial embedding is defined as follows:

$$H^{\left(0\right)}=[\begin{array}{c} 0\\ A^{T} \end{array}\;\begin{array}{c} A\\ 0 \end{array}].$$ (24)

The first layer of the GCN model embedding is calculated as follows:

$$H^{\left(1\right)}=\sigma \left(D^{-\frac{1}{2}}GD^{-\frac{1}{2}}H^{\left(0\right)}W^{\left(0\right)}\right),$$ (25)

where *W* ^(0)^ ∈ *R* ^(*M*+*N*)×*k*^ represents an input-to-hidden trained weight matrix, *H* ^(1)^ ∈ *R* ^(*M*+*N*)×*k*^ represents the first-layer propagation of features, including circRNAs and diseases. *K* denotes the embedding dimension in graph conventional layers. We adopt the exponential linear unit (Clevert et al., 2016) as the nonlinear activation function to enhance noise robustness and expressive capacity of the model in all graph convolutional layers. Eventually, the bilinear decoder *A′* proposed by Huang et al., (2020) is deployed to reconstruct the circRNA–disease association matrix as follows:

$$A^{\prime }=\mathrm{sigmoid}\left(H_{C}W^{\prime }H_{D}^{T}\right),$$ (26)

where *W′* ∈ *R* ^*k*×*k*^ denotes a trained weight matrix. *H*_*D*_ ∈ *R* ^*N*×*k*^ and *H*_*C*_ ∈ *R* ^*M*×*k*^ represent the last embedding for diseases and circRNAs, respectively. The final predicted relationship score *a′*_*ij*_ between circRNA *c*_*i*_ and disease *d*_*j*_ is obtained according to the corresponding (*i*, *j*)th entry of *A′*.

## Results

In this section, several verification experiments are deployed to assess the predictive capacity of GATGCN. First, we assess the influence of different parameters setting on GATGCN. Second, we introduce the evaluation metrics under leave-one-out cross-validation and 5-fold cross-validation to analyze the predictive capacity of GATGCN. Third, we design the ablation study to assess the impact of each part on GATGCN. Fourth, we discuss and compare GATGCN with state-of-the-art models on the same dataset. Last, case studies are deployed to further assess the performance in detecting potential relationships on GATGCN.

### Parameter Setting

**FIGURE 2:**
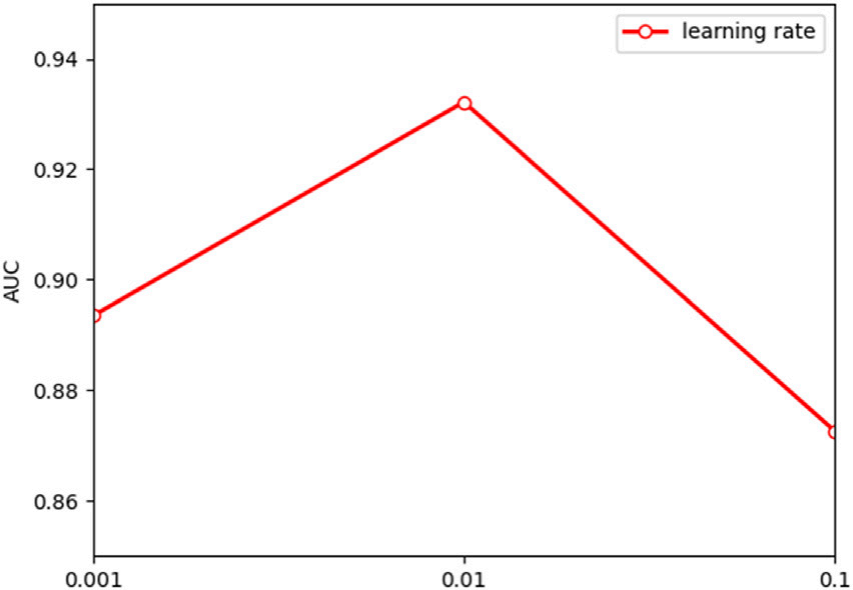
Outcome of comparing various learning rates.

**FIGURE 3:**
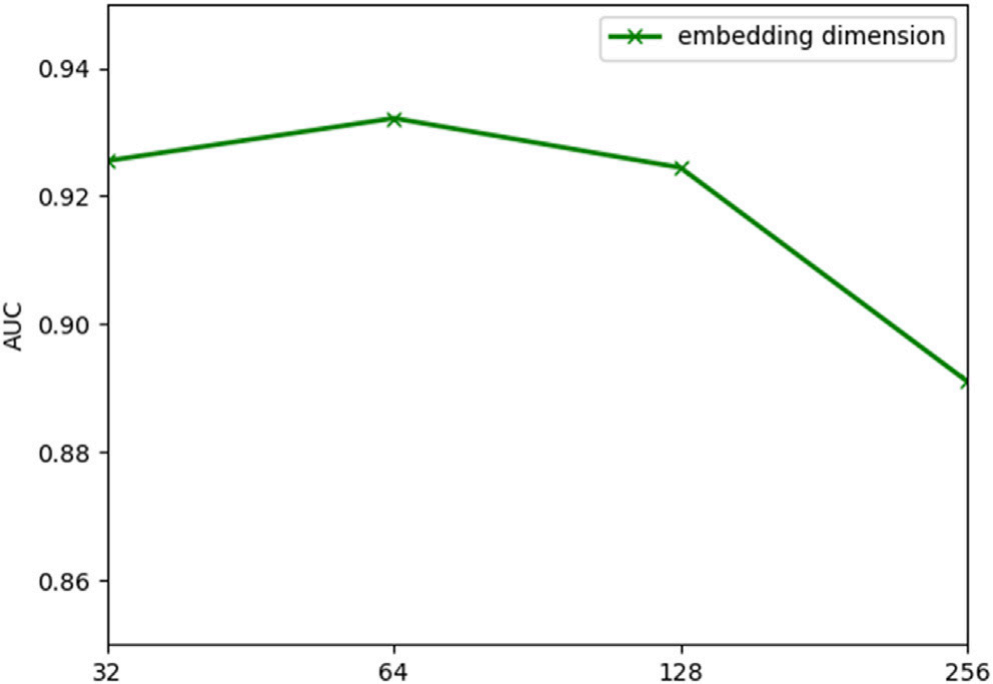
Outcome of comparing various embedding dimensions.

**FIGURE 4:**
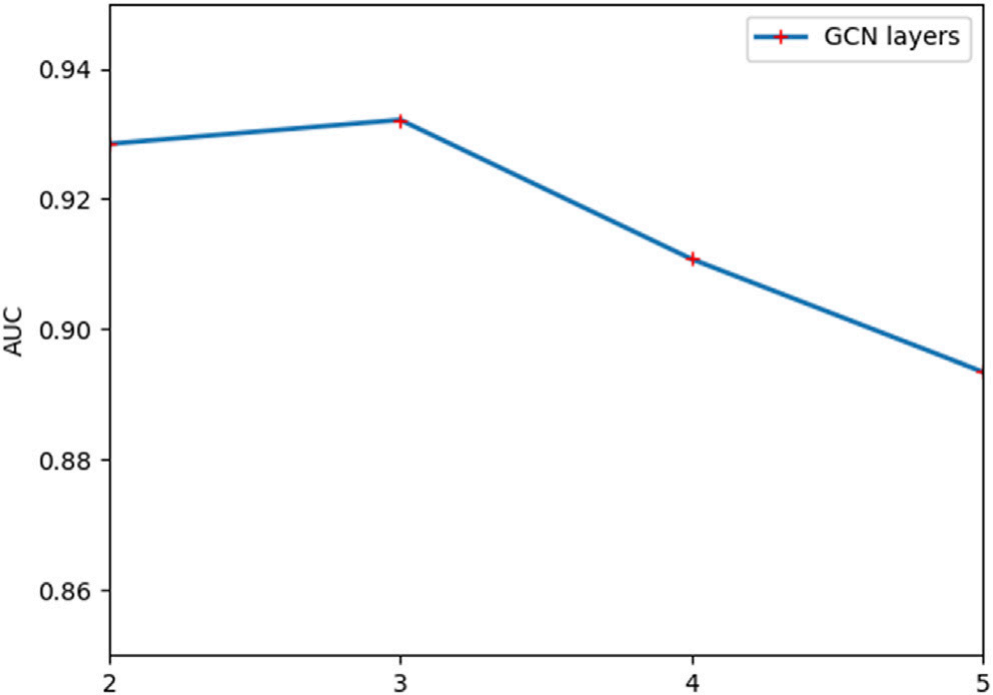
Outcome of comparing various GCN layers.

**FIGURE 5:**
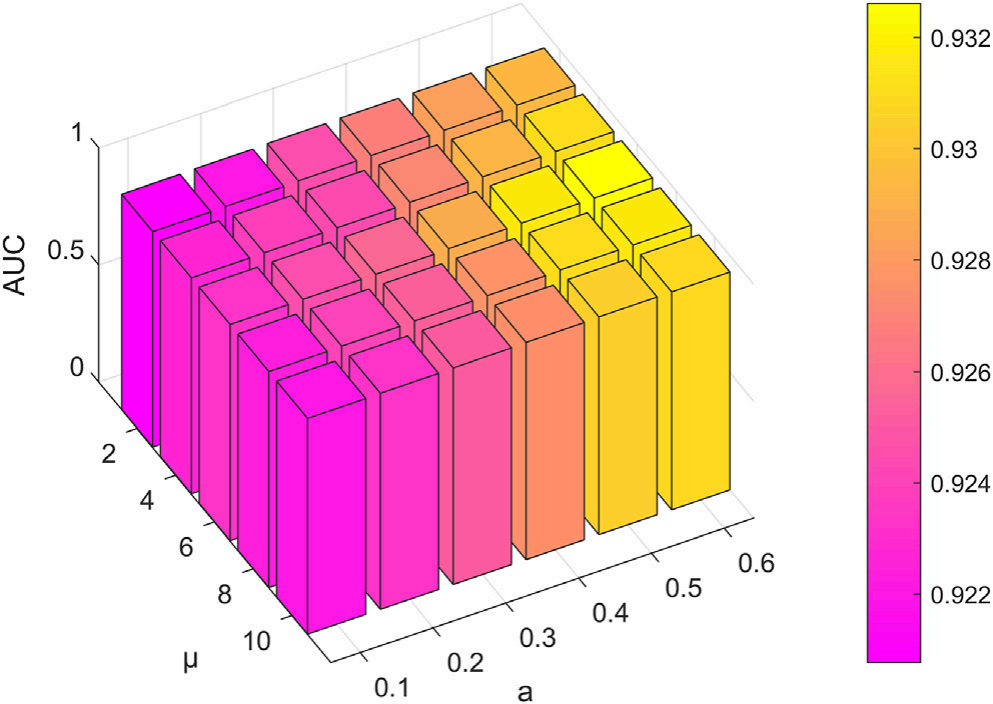
Outcome of comparing various dropout rates and penalty factors.

The performance of the model is frequently impacted by hyperparameter settings. Analysis of the parameters can quantitatively evaluate the stability of the model and provide a reference for parameter selection. The learning rate is significant to the convergence of the gradient descent algorithm in the model. Figure 2 indicates that the model will converge slowly with too small a learning rate, while too large a learning rate makes it hard to converge. According to the results in Figure 3, the embedding dimension within a certain size range has less impact on the convergence of our model. However, when the embedding dimension is too large, the model is prone to overfitting due to plenty of parameters. As shown in Figure 4, the model performs better with small layers of the graph convolutional network, and the performance drops significantly when the number of layers of GCN is *l* > 4. The reason is that the GCN with more layers not only captures more global prior information but also captures a lot of noise at the same time. Meanwhile, the penalty factor *µ* is set to regulate the contribution of learned similarity features in the propagation of convolutional layers, and the dropout rate *a* is adopted to avoid overfitting. As shown in Figure 5, the model achieves best performance at *µ* = 6 and *a* = 0.6.

### Evaluation Metrics

Cross-validation is a self-consistent testing approach widely adopted to demonstrate the predictive capacity of a method. The basic idea is to carry out the resampling method to select a portion of the benchmark data set as the training set to train the model, and the remaining samples to verify the model. Five-fold cross-validation and leave-one-out cross-validation are deployed to assess the predictive capacity of GATGCN. For five-fold cross-validation, the whole samples in the dataset are randomly separated into five roughly identical sections, four of which are adopted to train the GATGCN and the other is used to test the GATGCN. In order to decrease the bias produced by sample segmentation, the five-fold cross-validation is repeated 30 times to calculate the average result as the ultimate output. For leave-one-out cross-validation, each time only one sample in the dataset is selected among all recorded circRNA–disease relationships to test the model, and the remaining known relationships are utilized as training samples. In this study, since circRNA functional similarity relies on known associations; we recalculate the circRNA functional similarity in each repetition of the experiment.

In this study, the area under the curve (AUC) is applied as the primary metric to assess our model, which can visually show the predictive ability of GATGCN under each decision threshold. The basic principle is to treat the false-positive rate (FPR) and the true rate (TPR) as a two-dimensional coordinate point in a Cartesian coordinate system with FPR as the abscissa and TPR as the ordinate under different discrimination thresholds. Besides, several threshold-based metrics are adopted to further evaluate the predictive performance of the GATGCN including recall, specificity, accuracy, and F1. The detailed results of five-fold cross-validation and leave-one-out cross-validation are summarized in Table 1.

**TABLE 1:** Results generated by the GATGCN under five-fold CV and LOOCV.

Test set	Accu	Rec	Spe	F1	AUC
5-fold CV_1	0.988	0.682	0.989	0.437	0.956
5-fold CV_2	0.987	0.568	0.991	0.361	0.918
5-fold CV_3	0.987	0.644	0.988	0.373	0.922
5-fold CV_4	0.990	0.627	0.991	0.414	0.931
5-fold CV_5	0.991	0.647	0.990	0.402	0.934
Average	0.9886 ± 0.0024	0.6336 ± 0.0656	0.9898 ± 0.0012	0.3974 ± 0.0396	0.9322 ± 0.0238
LOOCV	0.987	0.782	0.992	0.542	0.951

### Ablation Study

**FIGURE 6:**
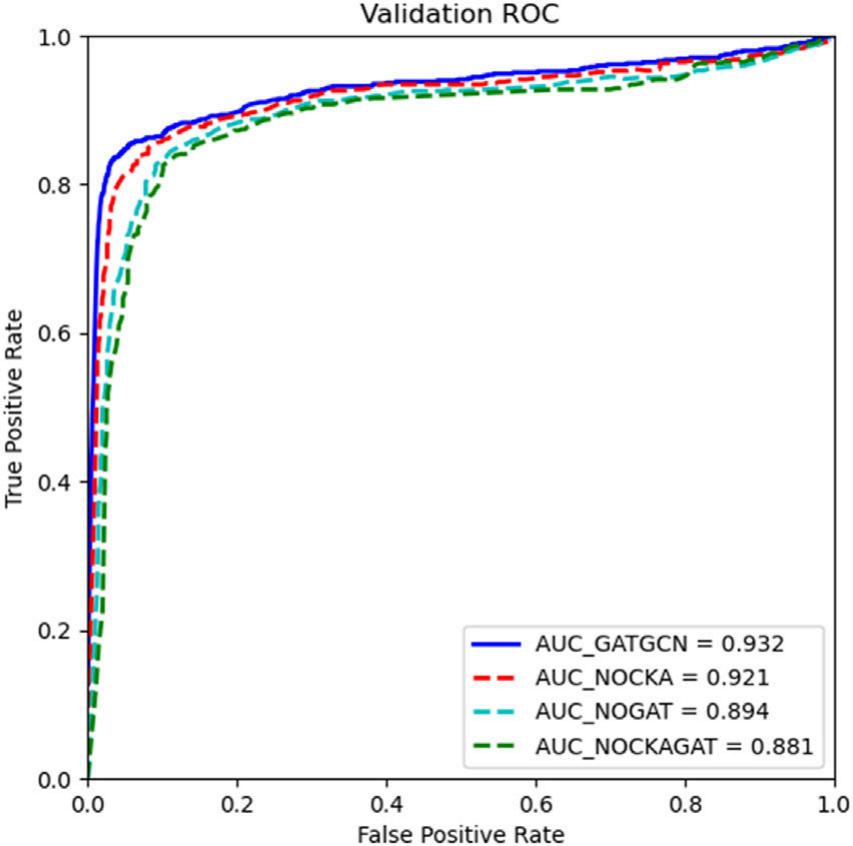
Performance of the GATGCN based on various model combinations.

The model GATGCN is used to detect potential relationships between diseases and circRNAs based on the centered kernel alignment model (CKA), graph attention network (GAT), and graph convolutional network (GCN). In order to verify the importance of CKA, GAT, and GCN in our model, we apply the ablation study to our model. In this part, we replace the CKA model with calculated average to fuse multisource similarity as NOCKA. Meanwhile, we only combine the CKA model and GCN model as NOGAT to calculate association scores. In addition, we only adopt the GCN to predict associations between diseases and circRNAs as NOCKAGAT. According to the results in Figure 6, the complete model GATGCN is compared with NOCKA, NOGAT, and NOCKAGAT with five-fold cross-validation, which achieves the best AUC of 0.932. In general, using the the graph attention network on the similarity network is beneficial to learn the latent representation of nodes. The AUC of GATGCN and NOCKA is significantly higher than that of the other two models, which indicates that GAT is significant to detect relationships between diseases and circRNAs. Moreover, the comparison between GATGCN and NOCKA suggests that the fusion of multisource similarity based on weights can improve performance in circRNA–disease relationship prediction.

### Comparison With Other Methods

**FIGURE 7:**
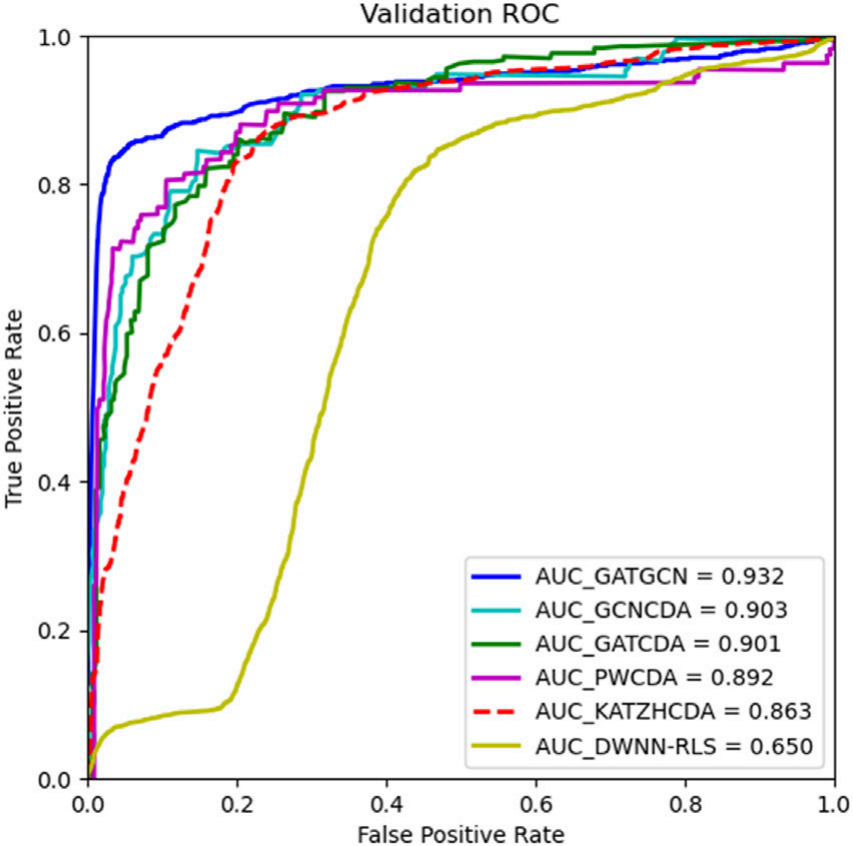
Comparison results of various prediction models under five-fold cross-validation.

To confirm the advantage of GATGCN, we compare it with several classic prediction methods with five-fold cross-validation. Since these methods adopt various datasets and evaluation metrics, we apply the same dataset and AUC as the metrics to compare the predictive capacity of models fairly and reasonably. In this part, the GATGCN is compared with several state-of-the-art methods, including KATZHCDA (Fan et al., 2018a), DWNN-RLS (Yan et al., 2018), PWCDA (Lei et al., 2018), GCNCDA (Wang et al., 2020b), and GATCDA (Bian et al., 2021). KATZHCDA is a graph-based method that uses the walking lengths and number of walks among nodes to measure the similarity among nodes in the heterogenous network. The DWNN-RLS measures initial relational values of new diseases and circRNAs *via* the decreasing weight k-nearest neighbor model and adopts the Kronecker product kernel to predict associations between diseases and circRNAs. The PWCDA predicts the circRNA–disease relationships by searching the paths without repeating for all circRNA–disease pairs based on the constructed heterogenous network. The GCNCDA extracts high-level features in the heterogenous network through graph convolutional neural networks and predicts the correlation between circRNAs and diseases *via* Forest by Penalizing Attributes. GATCDA learns the latent representation of nodes by assigning corresponding weights to each neighbor node, which efficiently aggregates the information of neighbor nodes and utilizes the local features of the graph. The results in Figure 7 indicate that GATGCN achieves the best AUC of 0.932, which is substantially greater than that of other models, and produces 7.9%, 43.3%, 4.5%, 3.2%, and 3.4% improvement in the AUC compared with KATZHCDA, DWNN-RLS, PWCDA, GCNCDA, and GATCDA respectively.

**FIGURE 8:**
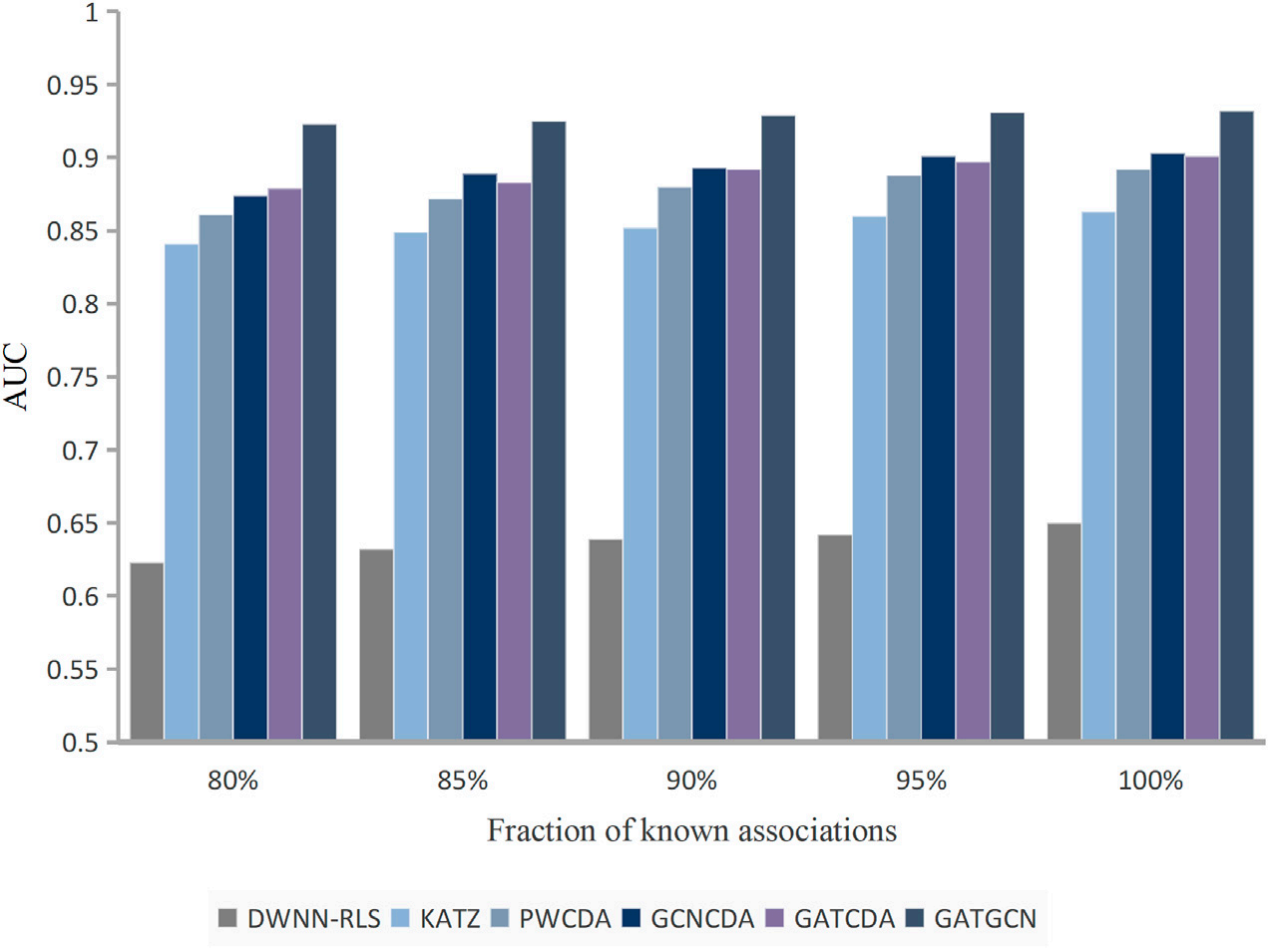
Performance of methods based on various percentages of known relationships.

Furthermore, the number of known interactions between diseases and circRNAs in the dataset can greatly affect the performance of the method, which also indicates the robustness of the method. Thus, we randomly remove a part of known associations between diseases and circRNAs at a ratio *r*∈{80%, 85%, 90%, 95%, and 100%} with five-fold cross-validation. As shown in Figure 8, the performance of GATGCN improves with increasingly known associations. Meanwhile, the GATGCN achieves the best result across different data richness among KATZ, DWNN-RLS, PWCDA, GCNCDA, and GATCDA.

### Case Studies

In this part, two kinds of case studies are utilized to further assess the reliability of the GATGCN for detecting potential circRNA–disease associations, which calculated the predicted probability matrix *via* a candidate set comprising unproven circRNAs. For the first kind of case study, all known circRNA–disease relationships are selected as training samples, and all unknown circRNA–disease relationships are prioritized according to the corresponding prediction scores. We select the top 10 scores by sorting the scores of the probability matrix in descending order and verified those predicted candidates through validated databases and literature, such as CircR2Disease, CircBase, and PubMed. Eventually, we adopt case studies on lung cancer, diabetes retinopathy, and prostate cancer.

Lung cancer occurs in the bronchial mucosa or glands with the highest incidence and the highest number of deaths in the world. The results in Table 2 show that six associations are verified by experiments among top 10 predicted candidate circRNAs for lung cancer. For example, the hsa_circ_0007385 (top 1) knockdown resulted in considerable inhibition of the proliferation, invasion, and migration of lung cancer cells (Jiang et al., 2018). Zhang et al. discovered that hsa_circ_0014130 (top 2) exhibited substantially overexpression in NSCLC tissues (Zhang S. et al., 2018). Zhu et al. indicated that hsa_circ_0016760 (top 3) accelerated the malignant growth of NSCLC by sponging miR-145-5p/FGF5 (Zhu et al., 2021).

**TABLE 2:** Top 10 candidate circRNAs related to lung cancer.

Rank	circRNA	Evidence (PMID)
1	hsa_circ_0007385	29372377
2	hsa_circ_0014130	29440731
3	hsa_circ_0016760	33416186
4	hsa_circ_0043256	28958934
5	hsa_circ_0012673	32141533
6	hsa_circRNA_404833	unconfirmed
7	hsa_circRNA_006411	unconfirmed
8	hsa_circRNA_401977	unconfirmed
9	hsa_circ_0013958	28685964
10	hsa_circ_0006404	unconfirmed

Diabetes retinopathy is a microvascular complication caused by diabetes, which can be divided into proliferative diabetic retinopathy and non-proliferative diabetic retinopathy. As shown in Table 3, the predictive results contain seven experimentally verified associations among the top 10 ranked candidate circRNAs. For instance, hsa_circRNA_063981 (top 1), hsa_circRNA_404457 (top 2), and hsa_circRNA_100750 (top 3) are considerably elevated in the serum of T2DR patients compared to T2DM and control patients (Gu et al., 2017).

**TABLE 3:** Top 10 candidate circRNAs related to diabetes retinopathy.

Rank	circRNA	Evidence (PMID)
1	hsa_circRNA_063981	28817829
2	hsa_circRNA_404457	28817829
3	hsa_circRNA_100750	28817829
4	hsa_circRNA_406918	28817829
5	hsa_circRNA_104387	28817829
6	hsa_circRNA_103410	28817829
7	hsa_circRNA_100192	28817829
8	hsa_circ_0013509	unconfirmed
9	circSLC8A1-1	unconfirmed
10	hsa_circ_101396	unconfirmed

Prostate cancer refers to malignant tumors produced by the epithelial cells of the prostate under the action of a variety of carcinogenic factors, which causes bone pain, pathological fractures, and paraplegia. Using the GATGCN, we successfully predict five of 10 top candidate circRNAs for prostate cancer (Table 4). The results in the literature indicate that circHIPK3 (top 1) expression is upregulated in prostate cancer cells and prostate cancer tissues (Liu et al., 2020). Kong et al. found that circFOXO3 (top 3) acted as a sponge for miR-29a-3p, exhibiting oncogenic activity in prostate cancer (Kong et al., 2020). Li et al. revealed that hsa_circ_0044516 (top 8) downregulation suppressed prostate cancer cell metastasis and growth (Li T. et al., 2020).

**TABLE 4:** Top 10 candidate circRNAs related to prostate cancer.

Rank	circRNA	Evidence (PMID)
1	circHIPK3	32547085
2	hsa_circ_0004383	unconfirmed
3	circ-Foxo3	31733095
4	hsa-circRNA 2149	unconfirmed
5	circR-284	unconfirmed
6	circDLGAP4	unconfirmed
7	hsa_circ_0008887	unconfirmed
8	hsa_circ_0044516	31625175
9	CDR1as	23900077
10	Cir-ITCH	32904490

In order to further assess the capacity of GATGCN for detecting new diseases, two common diseases, that is, clear cell renal cell carcinoma and cholangiocarcinoma are chosen for case studies. Specifically, all known associations about clear cell renal cell carcinoma and cholangiocarcinoma are reset to unknown and all candidate circRNAs are prioritized according to corresponding prediction scores. Eventually, we select the top 10 scores to assess the performance of GATGCN for detecting new circRNAs and diseases.

Cholangiocarcinoma is a malignant tumor that originates from the extrahepatic bile duct. The result in Table 5 shows that five associations are verified among the top 10 ranked candidate circRNAs. For example, Louis et al. demonstrated that the expression of circHIPK3 (top 2) was specifically elevated in cholangiocarcinoma cell lines (Louis et al., 2019). Chen et al. discovered that in cholangiocarcinoma, ciRS-7 (top 3) acts as an oncogene and promotes tumor development by competitively inhibiting miR-7. (Chen et al., 2021). Lu et al. indicated that circSMARCA5 (top 6) expression was lower in ICC tumor tissues than surrounding tissues (Lu and Fang, 2020).

**TABLE 5:** Top 10 candidate circRNAs related to cholangiocarcinoma.

Rank	circRNA	Evidence (PMID)
1	hsa_circ_000438	unconfirmed
2	circHIPK3	31654054
3	ciRS-7	33390857
4	circR-284	unconfirmed
5	circDLGAP4	unconfirmed
6	circSMARCA5	31880360
7	hsa_circ_0008887	unconfirmed
8	hsa_circ_0006404	unconfirmed
9	hsa_circRNA_000585	34182814
10	hsa_circ_0000673	33221765

Clear cell renal cell carcinoma is derived from adenocarcinoma of renal tubular epithelial cells, which forms hemangioma thrombus or metastasizes to lymph nodes and other organs. As shown in Table 6, the predicted results contain five experimental verified associations among the top 10 ranked candidate circRNAs. For example, Li et al. discovered that overexpression of circHIPK3 (top 1) substantially reduced CCRCC cell invasion and migration *in vitro* (Li H. et al., 2020). Zheng et al. discovered that circPVT1 (top 7) promotes progression in CCRCC cells by regulating TBX15 expression and sponging miR-145-5p (Zheng et al., 2021). Wang et al. indicated that hsa_circ_0001451 (top 8) upregulation could promote CCRCC cell invasion and proliferation (Wang G. et al., 2018).

**TABLE 6:** Top 10 candidate circRNAs related to clear cell renal cell carcinoma.

Rank	circRNA	Evidence (PMID)
1	circHIPK3	32409849
2	circR-284	unconfirmed
3	circDLGAP4	unconfirmed
4	hsa_circ_0004383	unconfirmed
5	Cir-ITCH	unconfirmed
6	hsa_circRNA_003251	unconfirmed
7	circPVT1	33453148
8	hsa_circ_0001451	30271486
9	ciRS-7	32496306
10	circZFR	31571906

The results of the case studies show that GATGCN can efficiently detect the potential circRNA–disease relationships and provide clues for exploring the mechanism between human complex diseases and circRNAs.

## Conclusion

Cumulative evidence has proved that the development of powerful calculation methods is significant to infer the interactions between diseases and circRNAs. These calculation models address challenges of high cost and high time consumption in conventional biological experiments. In this study, an advanced calculation method called GATGCN is designed to discover potential circRNA–disease relationships *via* graph attention mechanism and graph convolutional network. First, multisource similarity data for circRNAs and diseases are fused by the centered kernel alignment model. Second, the graph attention network is deployed to learn the dense representation of nodes on the disease–disease similarity network and circRNA–circRNA similarity network. Third, the heterogenous network is constructed by connecting known circRNA–disease associations, feature matrix of diseases, and feature matrix of circRNAs. Finally, the graph convolutional network is applied to get prediction scores based on the constructed heterogenous network. To further confirm the advantage of GATGCN for detecting circRNA–disease interactions, we compare it with several state-of-the-art prediction models under five-fold cross-validation. The results indicate that GATGCN achieves significant performance among compared methods. Meanwhile, the case study substantiates the excellent capability of the GATGCN for detecting potential circRNA–disease relationships. In conclusion, GATGCN is a powerful and promising approach for detecting circRNA–disease relationships.

Although we have integrated multisource biological information and utilized graph attention network and graph convolutional network to learn latent representation for diseases and circRNAs, there is still room to strengthen the predictive capability of the model. On the one hand, a large number of nonlinear features are extracted to detect circRNA–disease associations, which ignore the importance of linear features. We could further solve this problem by fusing nonlinear features and linear features to enhance the stability of our model. On the other hand, feature aggregation in excessive network layers could affect the expression of initial feature information. In the future, we can splice the representations of nodes in different layers as node features.

## Data Availability

The original contributions presented in the study are included in the article/Supplementary Material; further inquiries can be directed to the corresponding authors. The GATGCN dataset and code can be downloaded from https://github.com/ghli16/GATGCN.
